# Mapping Movement Behaviors and Mental Health Among Frontline Workers: Scoping Review

**DOI:** 10.2196/106002

**Published:** 2026-10-05

**Authors:** Amalie Skovgaard, Alison Kirk, Ellie Campbell, Gabrielle Horan-Buchanan, Nicola Cogan

**Affiliations:** 1 Department of Psychological Sciences and Health, University of Strathclyde, 40 George Street, Glasgow, Scotland, G1 1QE, United Kingdom, 44 0141 552 440; 2 Psychological Services, NHS Lanarkshire, Wishaw, United Kingdom

**Keywords:** frontline workers, mental health, 24-hour movement behaviors, sleep, physical activity, sedentary behavior, occupational health

## Abstract

**Background:**

Frontline workers experience substantial occupational demands that may affect sleep, physical activity, sedentary behavior, recovery, and mental health. Although 24-hour movement behavior (24hrMB) frameworks conceptualize these behaviors as interdependent components of a finite day, it remains unclear how extensively frontline research has adopted integrated approaches, or how evidence is distributed across behaviors, mental health domains, occupations, and measurement methods.

**Objective:**

This study aimed to map evidence examining movement behaviors and mental health among adult frontline workers; characterize its distribution and overlap across movement and mental health domains, occupational groups, methods, and contexts; and identify gaps for future research and intervention development.

**Methods:**

Following Joanna Briggs Institute methodology and PRISMA-ScR (Preferred Reporting Items for Systematic Reviews and Meta-Analyses extension for Scoping Reviews) guidance, 6 databases (PsycINFO, CINAHL, MEDLINE, SPORTDiscus, Scopus, and PubMed) were searched for peer-reviewed studies published from 2000 to 2026. The search was updated on July 30, 2026. Eligible studies included adult civilian frontline workers and examined at least 1 movement behavior in relation to at least 1 mental health domain. One reviewer (AS) screened and charted all studies, while 2 additional reviewers (GH-B and EC) independently screened and charted random subsets to assess reliability. Movement behaviors and mental health domains were coded as non–mutually exclusive. Findings were synthesized using descriptive statistics, narrative synthesis, and evidence mapping.

**Results:**

Across the original and updated searches, 613 studies were included. Research was concentrated among health care populations (n=468, 76.3%), with studies predominantly using cross-sectional (n=451, 73.6%), association-focused (n=526, 85.8%), and self-report movement measurement approaches (n=533, 86.9%). Sleep was assessed in 538 (87.8%) studies, physical activity in 177 (28.9%) studies, and sedentary behavior in 24 (3.9%) studies. Most studies assessed 1 movement behavior (n=500, 81.6%), and only 13 (2.1%) assessed all 3 behaviors. Stress was the most frequently assessed mental health domain (n=285, 46.5%), followed by depression (n=243, 39.6%), anxiety (n=224, 36.5%), burnout (n=154, 25.1%), and well-being (n=129, 21.0%). Evidence concentrated on sleep in relation to stress (n=248, 40.5%), depression (n=226, 36.9%), and anxiety (n=208, 33.9%), while comparatively little examined mental health alongside sedentary behavior. Only 9 (1.5%) studies used objective-only movement measurement.

**Conclusions:**

Despite a large and rapidly expanding literature, evidence remains dominated by sleep-focused, health care–based, cross-sectional, association-focused, and self-report research. Sedentary behavior, integrated 24hrMB approaches, non–health care frontline occupations, and longitudinal and objective methodologies remain underrepresented. By mapping movement and mental health domains as overlapping rather than mutually exclusive categories, this review identifies both major concentrations and substantive gaps in evidence. Future research should broaden occupational representation, use longitudinal and repeated-measures designs, and examine integrated 24hrMBs using complementary objective and subjective measures. In practice, current evidence can inform priorities for occupational monitoring and intervention development but is insufficient to support specific behavioral prescriptions.

## Introduction

### Background

Frontline occupations are characterized by high workload, trauma exposure, irregular schedules, shift work, emotional labor, and constrained opportunities for recovery, creating occupational conditions associated with elevated psychological burden [1,2]. Across health care, emergency response, policing, firefighting, and related frontline sectors, workers experience high levels of burnout, depression, anxiety, sleep disturbance, emotional exhaustion, and trauma-related symptoms [3-6]. Although the psychological impact of frontline work became increasingly visible during and following the COVID-19 pandemic, growing evidence suggests that these difficulties reflect broader occupational conditions, including sustained operational demands, circadian disruption, staffing pressures, and repeated stress exposure rather than pandemic-related burden alone [2,7,8]. Increasing attention has been directed toward 24-hour movement behaviors (24hrMBs), which conceptualize sleep, sedentary behavior, light physical activity, and moderate to vigorous physical activity as interdependent components of a finite 24-hour day [9,10]. This framework provides a useful perspective for examining health and well-being because occupational demands may influence how time is distributed across movement behaviors and opportunities for recovery [9,11]. Within frontline occupational settings, movement behaviors may be particularly relevant because irregular schedules, shift work, fatigue, and operational demands can constrain opportunities for recovery and create competing demands across the 24-hour day [1,2,8]. Understanding how movement behaviors are distributed and examined in relation to mental health within frontline occupational contexts may therefore provide important insight into the existing evidence base and identify opportunities for more integrated approaches to worker health and recovery [9,11].

### 24hrMBs and Mental Health in Frontline Work

Sleep disruption represents one of the most consistently examined behavioral pathways linking frontline occupational stress with mental health outcomes [2,3]. Many frontline roles involve night shifts, rotating schedules, extended working hours, on-call duties, and irregular operational demands that disrupt circadian rhythms and reduce opportunities for restorative sleep [8,12]. Poor sleep quality and insufficient sleep have been associated with depressive symptoms and perceived stress among frontline workers [13-15]. Physical activity has also received considerable attention in relation to mental health. Evidence from adult populations indicates that higher levels of physical activity are associated with better mental health and well-being [9,10]. Within frontline populations, physical activity may be particularly relevant because workers frequently experience elevated occupational stress and constrained opportunities for recovery [1,7,8,16,17]. However, movement patterns within frontline settings may be shaped by occupational demands, with some roles involving substantial physical exertion and others including prolonged periods of monitoring, driving, documentation, or administrative work [9,18]. Sedentary behavior represents a distinct movement behavior domain with potential relevance to frontline occupational research. Defined as waking behavior characterized by low energy expenditure while sitting or reclining, prolonged sedentary behavior has been associated with depressive symptoms and poorer mental well-being [10,12,19]. The distribution and potential implications of sedentary behavior may also vary according to occupational role structure and work demands [18,20]. Taken together, these behaviors form an interdependent 24-hour movement composition in which time allocated to 1 behavior necessarily affects the time available for others [9,11]. This interdependence highlights the value of examining movement behaviors within an integrated whole-day framework rather than solely as isolated behavioral domains [9,11]. However, the extent to which frontline occupational research has examined multiple movement behaviors concurrently or adopted approaches that account for their interdependence remains unclear. Mapping the representation and overlap of these behavioral domains is therefore necessary to establish how comprehensively the existing literature reflects an integrated 24hrMB perspective.

### Measurement Approaches in Frontline Research

Self-report measures provide practical and scalable methods for assessing behaviors across occupational populations, while objective approaches such as actigraphy, accelerometry, and wearable devices can provide repeated or continuous information on behavioral patterns in real-world environments [21,22]. These approaches may provide complementary information, as subjective and objective measures can capture different dimensions of sleep and movement behavior [21,23-25]. Advances in wearable and ambulatory technologies have expanded opportunities for objective monitoring within frontline occupational research [21,22,26-28]. However, the extent to which self-report, mixed, and objective movement measurement approaches have been adopted across frontline populations and movement behavior domains remains unclear. Mapping these approaches may help identify methodological concentrations and gaps within the current evidence base.

### Rationale for the Current Review

Despite increasing research examining movement behaviors and mental health among frontline workers, the literature spans diverse occupational populations, behavioral domains, mental health outcomes, study designs, measurement approaches, and occupational contexts. Consequently, the overall distribution and characteristics of this evidence base have not been comprehensively mapped. In particular, it remains unclear how research is distributed across sleep, physical activity, and sedentary behavior; which mental health domains are examined alongside these behaviors; how frequently multiple movement behaviors are assessed within the same study; and how evidence is distributed across frontline occupations, study designs, measurement approaches, and contexts. A scoping review is well suited to addressing these questions by systematically mapping the breadth, characteristics, and distribution of a heterogeneous evidence base [29-31]. Such an approach can identify areas of research concentration and relative scarcity across occupational populations, movement behaviors, mental health domains, and measurement approaches. This mapping can clarify the current state of the literature and identify methodological, occupational, behavioral, and conceptual priorities for future frontline occupational health research.

### Objectives and Review Questions

The primary objective of this scoping review was to systematically map the existing evidence examining movement behaviors and mental health domains among adult frontline workers. The secondary objectives were to (1) characterize the movement behavior and mental health domains examined across frontline populations; (2) describe the occupational groups, study designs, study contexts, and movement measurement approaches represented within the literature; (3) examine the distribution and overlap of movement behavior and mental health domains across the evidence base; and identify methodological, occupational, behavioral, and conceptual evidence gaps relevant to future research and intervention development.

The review sought to address the following questions: (1) What evidence exists examining movement behaviors and mental health domains among adult frontline workers? (2) Which frontline occupational groups, movement behavior domains, mental health domains, study designs, measurement approaches, and study contexts are represented within the literature? (3) How are movement behavior and mental health domains distributed and combined across the evidence base? (4) What methodological, occupational, and conceptual gaps are evident within the current literature?

## Methods

### Protocol and Registration

This scoping review followed Joanna Briggs Institute (JBI) methodology for scoping reviews and was reported in accordance with the PRISMA-ScR (Preferred Reporting Items for Systematic Reviews and Meta-Analyses extension for Scoping Reviews) guidelines [29,30]. A scoping review methodology was selected because the literature examining movement behaviors and mental health in frontline workers is heterogeneous in terms of occupational populations, behavioral operationalization, measurement approaches, study designs, and study contexts. Accordingly, the review aimed to map the breadth and characteristics of the evidence base rather than estimate pooled effect sizes or intervention effectiveness. A protocol outlining the review objectives, eligibility criteria, search strategy, screening procedures, charting framework, and synthesis approach was previously published in *JMIR Research Protocols* [32]. Methodological refinements and deviations from the published protocol are described in the Protocol Deviations subsection. A completed PRISMA-ScR checklist is provided in Checklist 1.

### Eligibility Criteria

Eligibility criteria were developed using the Population-Concept-Context framework [29,30]. Criteria relating to each component are described in the following sections.

### Population

Eligible studies included adults (aged ≥18 years) employed in civilian frontline occupations characterized by high occupational stress, trauma exposure, public-facing responsibilities, emergency response, or irregular operational demands. Populations included health care workers, emergency responders, firefighters, police officers, and related frontline occupational groups. Studies focusing exclusively on military populations, pediatric populations, or nonfrontline occupational groups were excluded unless frontline subgroup data could be extracted separately [6,33-35]. Military populations were excluded because deployment contexts and military organizational structures may introduce occupational demands that differ from those encountered in civilian frontline occupations [6]. Mixed-population studies were included only where data relating to eligible civilian frontline workers could be extracted separately; otherwise, they were excluded from the synthesis. During data charting, studies were categorized by occupational groups to facilitate comparison across frontline sectors.

### Concept

Eligible studies examined at least 1 movement behavior in relation to at least 1 mental health outcome. Movement behaviors included sleep, sedentary behavior, physical activity, and integrated 24hrMB frameworks. Mental health outcomes included stress, burnout, depression, anxiety, posttraumatic stress disorder (PTSD), or trauma-related symptoms, well-being, fatigue, suicidality, and other psychological constructs where authors explicitly framed outcomes as indicators of mental health or psychological functioning. To reduce conceptual heterogeneity, studies were included only where movement behaviors represented substantive variables of interest rather than incidental background characteristics.

### Context

Eligible studies were conducted within civilian frontline occupational environments characterized by high workload, shift work, emergency response, trauma exposure, or other operational stress. Studies conducted in clinical or workplace settings, as well as field, laboratory, or simulated occupational settings, were eligible provided they investigated movement behaviors and mental health among frontline worker populations. Comparative studies involving multiple frontline occupations or conducted across different countries were also eligible where they addressed the review objectives.

### Publication Characteristics

Studies published in English between 2000 and 2026 were eligible for inclusion. This time frame was selected to capture increasing adoption of wearable and ambulatory monitoring technologies alongside contemporary research examining movement behaviors and mental health in frontline workers [36,37]. Eligible evidence sources comprised peer-reviewed empirical quantitative, qualitative, and mixed methods studies reporting primary data. Reviews, editorials, commentaries, opinion pieces, and gray literature were excluded.

### Information Sources

Six electronic databases were searched: PsycINFO, CINAHL, MEDLINE, SPORTDiscus, Scopus, and PubMed. Studies published in English between 2000 and 2026 were eligible for inclusion. Gray literature was not systematically searched, and backward citation searching of reference lists and forward citation searching were not performed. Given the substantial volume of eligible literature identified, the review focused on mapping the peer-reviewed evidence base retrieved through the 6 bibliographic databases. The original searches were conducted on November 19, 2025, with updated searches conducted on July 30, 2026.

### Search

#### Search Overview

Search strategies were developed iteratively through exploratory searching, consultation with an academic librarian, and refinement of keywords and controlled vocabulary following JBI guidance and PRISMA-S (Preferred Reporting Items for Systematic Reviews and Meta-Analyses literature search extension) recommendations [29,38]. Search terms were organized into three principal concept blocks: (1) frontline workers and high-stress occupations, (2) movement behaviors and related measurement and monitoring terminology, and (3) mental health and psychological well-being. An additional exclusion block was used to reduce retrieval of clinical, patient, and military populations. The movement behavior concept incorporated terms relating to 24hrMBs, physical activity, sedentary behavior, sleep, and circadian or rest-activity patterns, alongside objective monitoring modalities and physiological or psychophysiological indicators that may be captured through wearable or sensor-based approaches (eg, heart rate, heart rate variability, electrodermal activity, respiratory measures, actigraphy, and accelerometry). These broader monitoring terms were included to maximize retrieval of potentially eligible studies in which movement behaviors were assessed alongside physiological monitoring, rather than to define physiological indicators as movement behaviors or mental health outcomes. Boolean operators (AND, OR, and NOT), truncation, phrase searching, and database-specific controlled vocabulary (where available) were used to optimize retrieval. Search strategies were adapted for each database to account for differences in indexing and syntax. Complete database-specific search strategies are provided in Multimedia Appendix 1, and the completed PRISMA-S checklist is provided in Checklist 2.

#### Selection of Sources of Evidence

All retrieved records were exported into EndNote [39] for deduplication before screening. Records were subsequently imported into Rayyan to support independent screening and duplicate verification [40]. Screening occurred across 3 stages: title screening, abstract screening, and full-text screening. One reviewer (AS) screened all records at each stage, while 2 additional reviewers (GH-B and EC) independently screened randomly selected subsets of records to assess screening reliability. Each additional reviewer independently screened a randomly selected subset of approximately 2%‐5% of records at each stage, resulting in up to 10% overlap with the primary reviewer. Percentage agreement was calculated separately at each screening stage across records independently assessed by both the primary reviewer and additional reviewers. During title and abstract screening, an inclusion-favoring approach was adopted whereby records marked as “include” or “unsure” by either reviewer progressed to the next stage. At full-text screening, studies were required to meet all Population-Concept-Context eligibility criteria for inclusion. Prior to the main screening, pilot screening exercises were conducted at each stage to standardize the interpretation and application of the eligibility criteria [41,42]. Initial title-screening agreement was 84%. Following discussion and refinement of the screening guidance, a second title-screening pilot achieved 94% agreement. Subsequent pilot exercises achieved 100% agreement on both abstract and full-text screening. During the main screening, agreement across independently screened subsets was 99% at title screening, 90% at abstract screening, and 91% at full-text screening. Disagreements were resolved through discussion and consensus, with third reviewer adjudication available where required. The updated search was screened using the same eligibility criteria and screening procedures. Agreement across independently screened subsets was 97% at title screening and 100% at both abstract and full-text screening.

#### Data-Charting Process

A structured charting framework was developed in accordance with JBI guidance and refined through pilot testing. The initial charting framework was developed a priori to capture study characteristics, movement behaviors, mental health domains, measurement approaches, and contextual factors relevant to the review objectives, and was iteratively refined during charting to accommodate relevant characteristics identified within the included literature. The charting form was piloted independently on a subset of studies to assess clarity, completeness, and consistency of the extraction framework. Any discrepancies or ambiguities identified during piloting were discussed and resolved before full data charting commenced. One reviewer (AS) charted data from all included studies. Two additional reviewers (GH-B and EC) independently charted randomly selected subsets of approximately 2%‐5% of included studies each, resulting in up to 10% of studies undergoing independent duplicate charting to assess reliability. Agreement was calculated across studies independently charted by the primary and additional reviewers, with 100% agreement achieved. Discrepancies, if identified, were resolved through discussion and consensus, with consultation from an additional reviewer where required.

### Data Items

Data items charted included publication year, country, frontline occupational group, study design, sample characteristics, movement behavior domains, movement measurement approach, mental health domains, mental health measurement instruments, study focus, study context, and key findings. Study design and study focus were coded as separate dimensions. Study design described the methodological structure of the study (eg, cross-sectional, longitudinal, intervention, experimental, mixed methods, or qualitative), whereas study focus described the principal analytic purpose of the study (association, intervention, or monitoring). Consequently, counts within these 2 classification schemes were not expected to correspond directly. Movement behaviors and mental health domains were charted as non–mutually exclusive categories. Movement behaviors and mental health domains were coded as present when directly assessed within a study, irrespective of whether they were analyzed as primary exposures, outcomes, covariates, or descriptive variables. Where studies assessed multiple movement behaviors or mental health domains, each relevant category was coded independently. The sleep domain included measures of sleep duration, sleep quality, sleep efficiency, sleep latency, wake after sleep onset, sleep regularity, chronotype, insomnia, and other sleep-related characteristics. Sleep measures were categorized within this common domain for evidence-mapping purposes while retaining their original study-level operationalization. Divergent sleep metrics were not quantitatively pooled or treated as equivalent measures. The physical activity domain included assessments of physical activity or exercise, including frequency, duration, intensity, steps, and energy expenditure, when used as a proxy for activity. The sedentary behavior domain included sitting time, sedentary time, sedentary bouts, screen-based sedentary behavior, and related sedentary measures.

Mental health domains included stress, burnout, depression, anxiety, PTSD or trauma, well-being, fatigue, suicidality, and other mental health outcomes. Where studies reported multiple mental health domains, each relevant domain was coded independently. The well-being domain included measures explicitly assessing psychological or mental well-being, flourishing, or life satisfaction. Generic health-related quality-of-life measures were not classified as well-being unless they specifically assessed psychological well-being. The “Other Mental Health” category captured psychological constructs that did not align with the predefined domains, including fear, affect, resilience, and related constructs. Movement measurement approach was charted separately from the movement behavior assessed. Approaches were classified according to whether movement behaviors were assessed using self-report, objective measurement, or a combination of subjective and objective methods. Objective approaches were further characterized according to the measurement modality reported, including wearable devices, accelerometry, and actigraphy. Psychophysiological indicators were included within the search strategy to maximize retrieval of studies using objective or wearable monitoring. Individual physiological signals and derived psychophysiological metrics were not independently charted as analytic domains and did not contribute to the movement behavior or mental health domain frequency counts or evidence maps. Both subjective and objective movement measurement approaches were eligible because the review aimed to map the full range of measurement practices used across frontline research rather than privilege or restrict inclusion to a single modality. This allowed the distribution of self-report, objective, and mixed approaches to be examined without treating these approaches as methodologically equivalent or pooling their estimates.

### Synthesis of Results

Findings were synthesized descriptively using narrative synthesis, summary tables, and evidence-mapping approaches [29,43]. Studies were grouped according to frontline occupational sector, movement behavior domain, mental health outcome domain, measurement approach, study design, and contextual setting. Descriptive frequencies and percentages were calculated to summarize the distribution of study characteristics, movement behaviors, mental health domains, and measurement approaches across the evidence base. Because movement behavior and mental health domains were coded as non–mutually exclusive, individual studies could contribute to multiple categories. Consequently, frequencies and percentages across these domains were not expected to sum to the total number of included studies or 100%. Evidence mapping was used to examine the distribution and intersection of movement behaviors, mental health domains, occupational groups, and measurement approaches. Evidence gap maps were developed to visually identify areas of research concentration and relative scarcity across these dimensions. The synthesis aimed to identify evidence clusters, methodological trends, underrepresented occupational groups, and conceptual gaps relevant to future research and intervention development. No meta-analysis or pooled effect estimation was undertaken because of substantial heterogeneity in study populations, designs, movement and mental health measures, and analytical approaches. Formal critical appraisal was not conducted because the objective of the review was to map the breadth and characteristics of the evidence base rather than assess study quality or estimate pooled effects. Methodological limitations identified across the evidence base, including patterns in study design, measurement approach, and monitoring methodology, were documented narratively during synthesis.

### Protocol Deviations

Several methodological refinements and deviations were made following publication of the protocol [32]. Movement behavior and mental health outcome categories were revised from the mutually exclusive classification originally specified in the protocol to non–mutually exclusive coding, allowing studies assessing multiple behaviors to contribute to each relevant domain. Mental health outcome categories were also iteratively refined during data charting to reflect the range of psychological constructs identified across the included literature. The planned synthesis approach was refined to incorporate descriptive evidence mapping and evidence gap visualizations alongside narrative synthesis and summary statistics. Formal thematic analysis of qualitative evidence, as proposed in the protocol, was not undertaken because the final review focused on mapping the distribution, measurement, and characteristics of the evidence base rather than synthesizing qualitative experiences as a distinct body of evidence. The screening and data-charting procedures were also refined from those described in the protocol. Rather than all records being independently assessed or charted by multiple reviewers, 1 reviewer (AS) completed screening and data charting for all eligible records, with 2 additional reviewers (GH-B and EC) independently assessing randomly selected subsets to evaluate reliability. Disagreements were resolved through discussion and consensus, with additional adjudication where required. The literature search was additionally updated on July 30, 2026, to capture studies published since completion of the original search. Correspondingly, the eligible publication period was extended from 2000 to 2025 to include studies published in 2026.

The same eligibility criteria and screening procedures were applied to newly identified records. Although targeted gray literature searching was specified in the published protocol, this was not undertaken in the final review. Given the substantial volume of eligible peer-reviewed literature identified through the database searches, the final review focused on systematically mapping the peer-reviewed evidence base. The final database set also differed from that described in the published protocol. ScienceDirect was not retained as a separate database source in the final search, and the completed review reports on the 6 bibliographic databases ultimately searched: PsycINFO, CINAHL, MEDLINE, SPORTDiscus, Scopus, and PubMed. Collectively, these methodological refinements were implemented to improve the comprehensiveness and accuracy of the final evidence mapping and to ensure that the synthesis appropriately reflected the breadth, overlap, and characteristics of the evidence identified during the review.

### Stakeholder Consultation

Stakeholder consultation was incorporated throughout the review to support the relevance, applicability, and interpretation of findings within frontline occupational settings. Stakeholders included health care professionals, occupational health researchers, and representatives from organizations supporting frontline worker well-being, and experts in digital mental health and occupational monitoring. Early consultation informed refinement of the review questions, eligibility criteria, and search strategy, including the incorporation of psychophysiological monitoring terminology. Following data charting, stakeholders supported interpretation of emerging patterns, methodological considerations, evidence gaps, and the feasibility of self-report and objective monitoring approaches. Consultation was conducted through meetings, discussions, and email correspondence. Consistent with JBI guidance, this process strengthened the contextual relevance and applicability of the review findings [29].

### Ethical Considerations

Ethical approval was not required because the review synthesized existing publicly available literature and did not involve primary data collection or direct involvement of human participants.

## Results

### Study Selection

The original search identified 18,069 records through database searching. After removal of duplicates, 9874 records were retained for title screening. Following title screening, 3616 records progressed to abstract screening, of which 2892 were retained for full-text assessment. After full-text screening, 527 studies met the inclusion criteria and were included in the review. The study selection process for the original search is summarized in Figure 1.

The literature search was updated on July 30, 2026, to identify studies published since completion of the original search. The updated search identified 2372 records. Following duplicate removal, 1395 records were retained for title screening. Of these, 307 progressed to abstract screening and 281 underwent full-text assessment. Following full-text screening, 86 additional studies met the inclusion criteria. Across the original and updated searches, 613 studies were included in the final scoping review.

**Figure 1.**
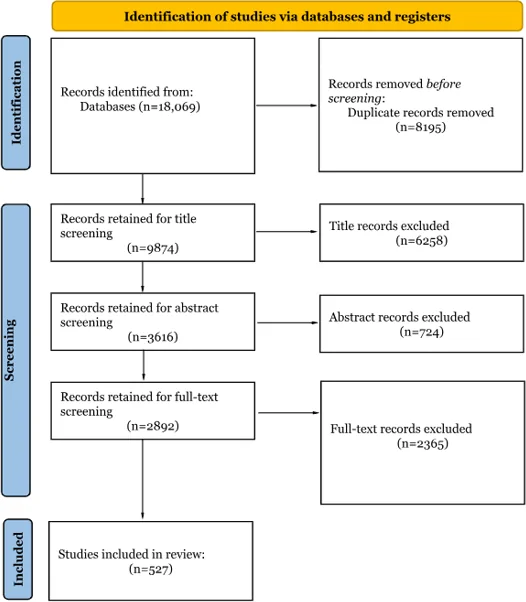
PRISMA-ScR (Preferred Reporting Items for Systematic Reviews and Meta-Analyses extension for Scoping Reviews) flow diagram of study identification, screening, eligibility assessment, and inclusion for the original search.

### Characteristics of Included Studies

The 613 included studies were published between 2002 and 2026, despite eligibility extending to studies published from 2000 onward, with a marked increase in publications in recent years (Table 1). Only 10 (1.6%) studies were published between 2000 and 2009 and 104 (17.0%) studies between 2010 and 2019, whereas 499 (81.4%) studies were published from 2020 onward. The largest number of studies was published in 2025 (n=125, 20.4%), followed by 2021 (n=84, 13.7%), 2022 (n=73, 11.9%), 2023 (n=73, 11.9%), and 2024 (n=72, 11.7%). Studies represented a broad international evidence base. Following harmonization of obvious country label variants in the charting data, China contributed the largest number of studies (n=108, 17.6%), followed by the United States (n=104, 17.0%), Turkey (n=34, 5.5%), Brazil (n=26, 4.2%), South Korea (n=22, 3.6%), Taiwan (n=22, 3.6%), and Australia (n=20, 3.3%). Studies were also conducted across Europe, Asia, the Middle East, Africa, North and South America, and multinational settings. Health care workers represented the majority of the evidence base (n=468, 76.3%). Police and law enforcement personnel accounted for 55 (9.0%) studies, firefighters for 42 (6.9%) studies, paramedics and emergency medical services personnel for 25 (4.1%) studies, and mixed frontline worker populations for 23 (3.8%) studies. Cross-sectional studies predominated (n=451, 73.6%), followed by longitudinal studies (n=71, 11.6%) and intervention studies (n=54, 8.8%). Mixed methods (n=15, 2.4%), experimental (n=14, 2.3%), and qualitative studies (n=8, 1.3%) were comparatively uncommon. Most studies were classified as association studies (n=526, 85.8%), while 68 (11.1%) were intervention studies and 19 (3.1%) were monitoring studies. Regarding study context, 430 (70.1%) studies were conducted under routine or noncrisis conditions, while 175 (28.5%) studies were conducted in the context of a pandemic. A further 6 (1.0%) studies were conducted in other crisis contexts and 2 (0.3%) studies in the context of natural disasters.

**Table 1.** Characteristics of included studies across frontline occupational groups, study designs, study focus, and contextual factors (N=613)^a^.

Characteristics	Studies
Frontline occupational group, n (%)	
Health care workers	468 (76.3)
Police or law enforcement	55 (9)
Firefighters	42 (6.9)
Paramedics or emergency medical services	25 (4.1)
Mixed frontline workers	23 (3.8)
Study design, n (%)	
Cross-sectional	451 (73.6)
Longitudinal	71 (11.6)
Intervention	54 (8.8)
Mixed methods	15 (2.4)
Experimental	14 (2.3)
Qualitative	8 (1.3)
Study focus, n (%)	
Association study	526 (85.8)
Intervention study	68 (11.1)
Monitoring study	19 (3.1)
Study context, n (%)	
Routine	430 (70.1)
Pandemic	175 (28.5)
Other crisis	6 (1)
Natural disaster	2 (0.3)

a Percentages are based on the total number of included studies (N=613) and may not sum to exactly 100% because of rounding.

### Movement Behavior Domain

Movement behaviors were coded as non–mutually exclusive domains, allowing studies assessing more than 1 behavior to contribute to each relevant category. Of 613 studies, sleep was the most frequently examined movement behavior assessed in 538 (87.8%) studies. Physical activity was assessed in 177 (28.9%) studies, while sedentary behavior was examined in only 24 (3.9%) studies. As studies could assess more than 1 movement behavior, these percentages do not sum to 100%. Most studies focused on a single behavior. In total, 500 (81.6%) studies assessed only 1 movement behavior, compared with 113 (18.4%) studies that assessed 2 or more. Sleep alone accounted for 433 (70.6%) studies, while physical activity alone was examined in 66 (10.8%) studies and sedentary behavior alone in only 1 (0.2%) study. Among studies examining multiple behaviors, 90 (14.7%) assessed sleep and physical activity, 8 (1.3%) assessed physical activity and sedentary behavior, and 2 (0.3%) assessed sleep and sedentary behavior. Only 13 (2.1%) studies assessed all 3 movement behaviors, sleep, physical activity, and sedentary behavior, within the same study (Figure 2).

**Figure 2.**
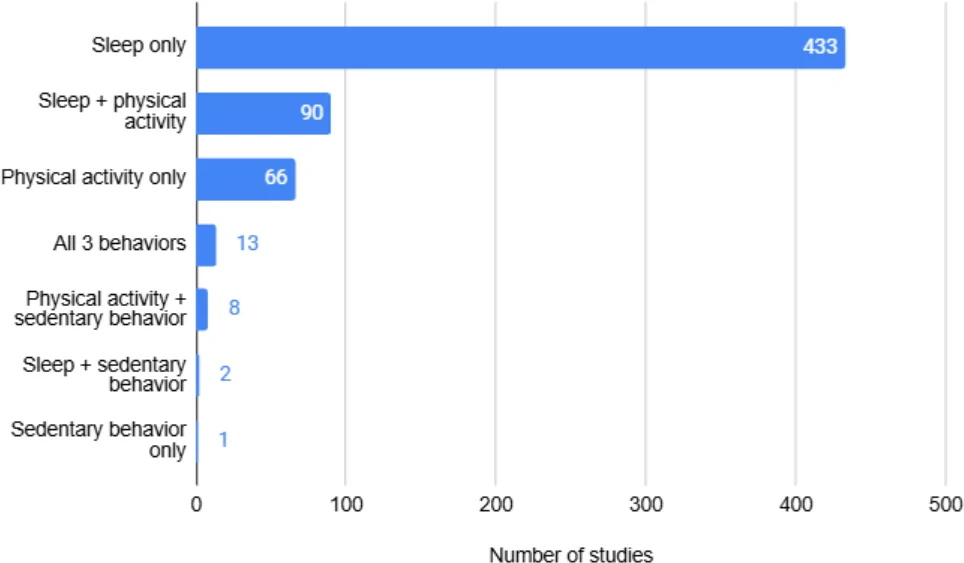
Distribution and overlap of movement behaviors assessed across included studies (N=613). Movement behavior domains were coded independently and were non–mutually exclusive*.* The categories displayed in the figure represent mutually exclusive combinations derived from these domains. “All 3 behaviors” refers to studies assessing sleep, physical activity, and sedentary behavior within the same study.

### Mental Health Domains

Mental health outcomes were coded as non–mutually exclusive domains, allowing studies assessing multiple psychological outcomes to contribute to each relevant category (Figure 3). Stress was the most frequently assessed mental health outcome, examined in 46.5% (285/613) of the studies, followed by depression (n=243, 39.6%) and anxiety (n=224, 36.5%). Burnout was assessed in 154 (25.1%) studies, while well-being was examined in 129 (21.0%). PTSD or trauma-related was assessed in 76 (12.4%) studies and fatigue in 59 (9.6%) studies. A further 42 (6.9%) studies assessed other mental health domains, encompassing psychological constructs that did not align with the predefined mental health domains, while suicidality was assessed in 14 (2.3%) studies. As individual studies could assess multiple mental health domains, percentages across domains do not sum to 100%.

**Figure 3.**
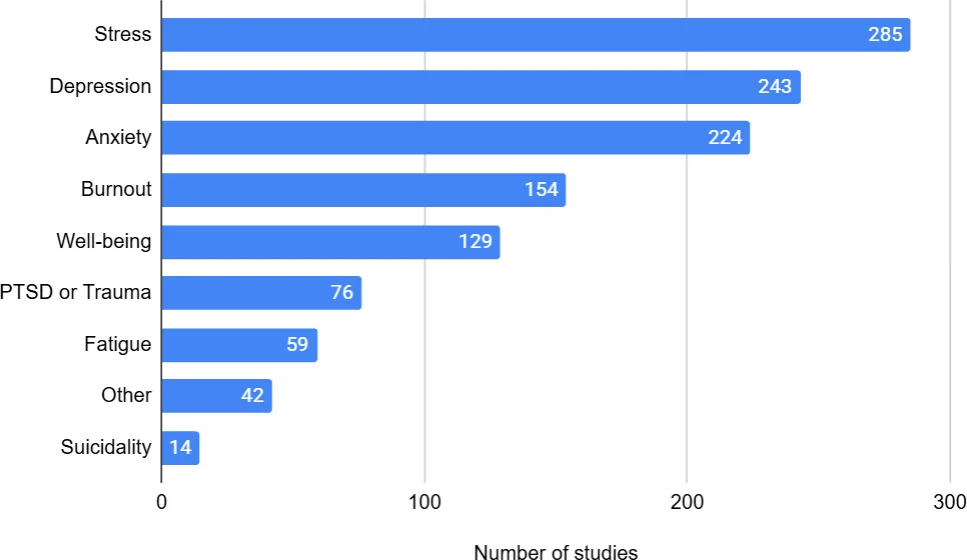
Distribution of mental health domains assessed across included studies (N=613). Mental health domains were coded independently and were non–mutually exclusive. Individual studies could therefore contribute to more than 1 domain. Accordingly, frequencies and percentages across domains do not sum to the total number of included studies or 100%. PTSD: posttraumatic stress disorder.

### Movement Behaviors and Mental Health Domains

Evidence mapping showed substantial variation in the volume of research across movement behavior and mental health domain combinations (Figure 4). The largest concentrations of evidence involved sleep in relation to stress (n=248), depression (n=226), and anxiety (n=208), followed by sleep in relation to burnout (n=130) and well-being (n=106). Among studies assessing physical activity, stress was the most frequently examined mental health domain (n=89), followed by depression (n=65), anxiety (n=61), and well-being (n=55). Comparatively fewer studies examined sedentary behavior in relation to mental health domains. The most frequently represented combinations were sedentary behavior with stress (n=14), anxiety (n=11), and depression (n=10), while each remaining mental health domain was represented in fewer than 10 studies. Overall, the evidence gap map demonstrated that research was concentrated on sleep in relation to stress, depression, and anxiety, with substantially fewer studies examining physical activity and particularly sedentary behavior across mental health domains.

**Figure 4.**
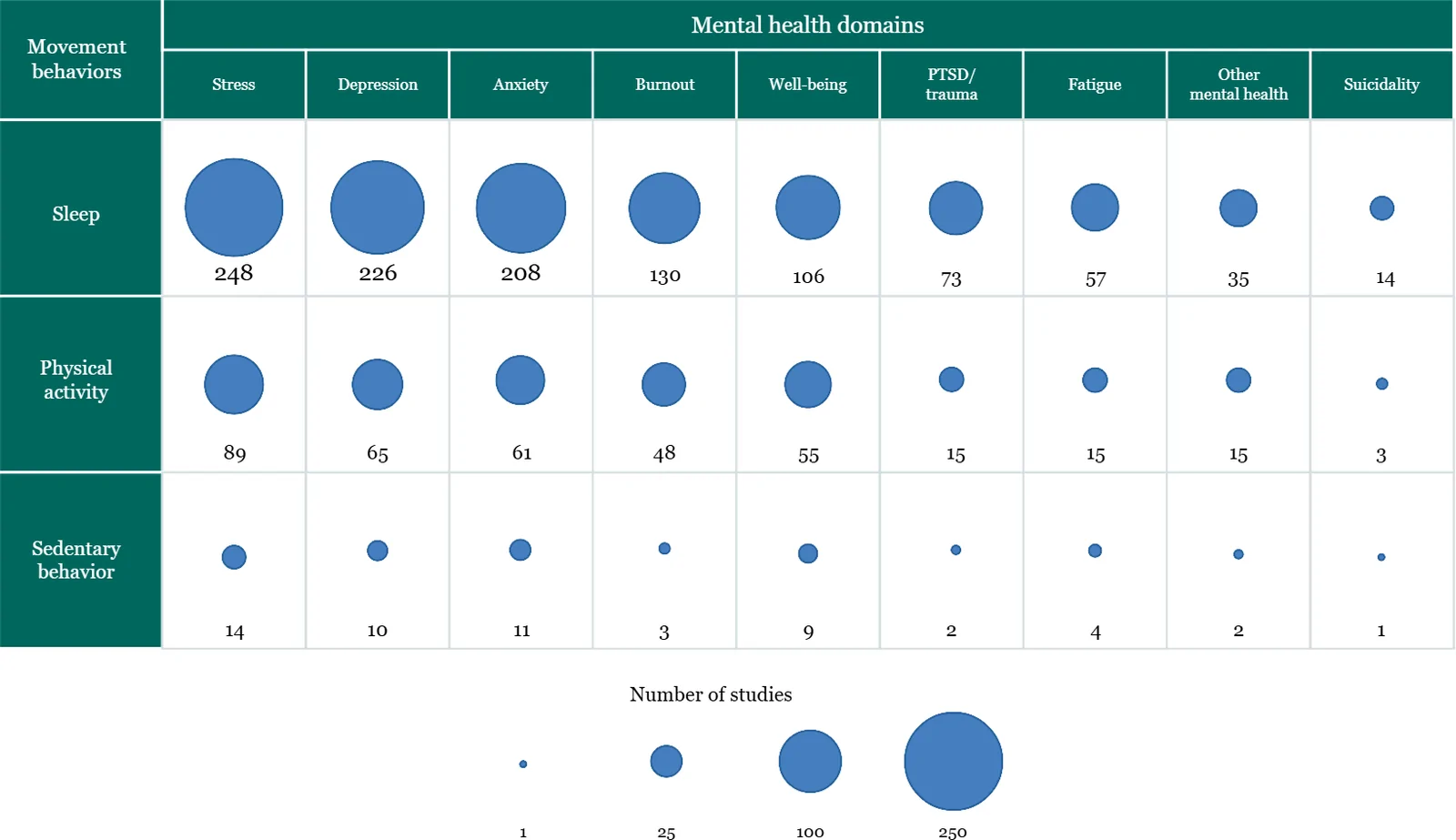
Bubble evidence gap map of movement behaviors and mental health domains across included studies (N=613). Movement behaviors and mental health domains were coded independently and were non–mutually exclusive. Individual studies could therefore contribute to multiple movement behavior and mental health domain combinations. Bubble area represents the number of included studies assessing each combination and reflects the volume of evidence rather than the strength of evidence. “Other mental health” includes fear, affect, resilience, and related constructs. PTSD: posttraumatic stress disorder.

### Measurement Approaches

Self-report measures predominated across the evidence base, representing the movement measurement approach in 86.9% (533/613) of studies. Mixed measurement approaches, incorporating both self-report and objective methods, were used in 71 (11.6%) studies. Objective-only measurement approaches were comparatively uncommon: 7 (1.1%) studies were classified as using wearable devices, 1 (0.2%) used accelerometry, and 1 (0.2%) used actigraphy. Overall, only 9 (1.5%) studies relied exclusively on an objective movement measurement approach, although objective measures were additionally incorporated within the 71 studies classified as using mixed measurement approaches. Measurement approaches also varied across movement behavior domains (Table 2). Among the 538 studies assessing sleep, 476 (88.5%) used self-report measures, while 55 (10.2%) used mixed measurement approaches. Objective-only approaches were uncommon, comprising 6 (1.1%) studies using wearable devices and 1 (0.2%) study using actigraphy. Among the 177 studies assessing physical activity, 133 (75.1%) used self-report measures and 39 (22.0%) used mixed measurement approaches, while 4 (2.3%) used wearable devices and 1 (0.6%) used accelerometry. Of the 24 studies assessing sedentary behavior, 12 (50.0%) used self-report measures, 10 (41.7%) used mixed measurement approaches, and 2 (8.3%) used wearable devices. Self-report remained the predominant measurement approach across all 3 movement behavior domains. However, mixed and objective measurement approaches represented a greater proportion of the comparatively small sedentary behavior literature.

**Table 2.** Movement measurement approaches according to movement behavior domain^a^.

Movement behavior	Self-report	Mixed	Wearable device	Accelerometer	Actigraphy	Total
Sleep	476 (88.5%)	55 (10.2%)	6 (1.1%)	0 (0.0%)	1 (0.2%)	538
Physical activity	133 (75.1%)	39 (22%)	4 (2.3%)	1 (0.6%)	0 (0.0%)	177
Sedentary behavior	12 (50%)	10 (41.7%)	2 (8.3%)	0 (0.0%)	0 (0.0%)	24

a Values are n (% within movement behavior domain). Movement behavior domains were coded independently and were non–mutually exclusive. Individual studies could therefore contribute to more than 1 row. Measurement approaches reflect the categories applied during data charting. “Mixed” refers to studies incorporating both self-report and objective movement measurement approaches.

### Evidence Gaps and Research Concentrations

Taken together, the evidence base was concentrated within health care populations, cross-sectional and association-focused designs, sleep research, and self-report measurement. Comparatively limited evidence examined sedentary behavior, integrated movement behavior approaches, objective measurement methods, or frontline occupational groups outside health care. Evidence was also unevenly distributed across movement behavior and mental health combinations, with the strongest concentrations involving sleep alongside stress, depression, and anxiety. These patterns identify clear behavioral, occupational, and methodological gaps for future research.

## Discussion

### Principal Findings

This scoping review mapped the current evidence examining movement behaviors and mental health among frontline workers and identified a large but unevenly distributed literature. Across 613 studies, evidence was concentrated within health care populations, cross-sectional and association-focused designs, sleep research, and self-report measurement. Physical activity and particularly sedentary behavior received substantially less attention. Although some studies examined multiple movement behaviors, only 13 examined sleep, physical activity, and sedentary behavior together, indicating limited representation of integrated 24hrMB approaches. Mental health research was similarly concentrated around stress, depression, and anxiety, particularly in relation to sleep. Taken together, the findings demonstrate substantial growth in this field while revealing persistent behavioral, occupational, and methodological imbalances.

### Movement Behavior Domains

Sleep was the dominant movement behavior across the evidence base, assessed in 87.8% (538/613) of the studies. This concentration likely reflects the long-standing relevance of sleep to frontline occupational health. Frontline work frequently involves irregular schedules, night shifts, extended working hours, high occupational demands, and restricted opportunities for recovery, all of which can disrupt sleep and circadian functioning [1-3,7,8]. Given the established importance of sleep for recovery, emotional regulation, and occupational functioning, its prominence within the literature is therefore unsurprising [2,13-15]. The prominence of sleep-focused research may also reflect the relevance of circadian disruption within frontline work, as irregular and night shift schedules can alter sleep-wake timing, sleep, and recovery opportunities [2]. Nevertheless, the extent of this concentration indicates that current understanding of movement behaviors and mental health among frontline workers is largely shaped by sleep-focused research. Physical activity was examined considerably less frequently, appearing in 177 (28.9%) studies, while sedentary behavior was assessed in only 24 (3.9%). The particularly limited representation of sedentary behavior constitutes an important gap because sedentary behavior is a distinct component of daily movement rather than simply the absence of physical activity [10]. Greater attention to sedentary behavior may therefore contribute to a more complete understanding of how occupational movement patterns relate to mental health and well-being across different frontline settings.

The limited integration of movement behaviors within individual studies represents a further conceptual gap. Although 113 (18.4%) studies assessed 2 or more movement behaviors, only 13 (2.1%) assessed sleep, physical activity, and sedentary behavior within the same study. Importantly, concurrent assessment of multiple behaviors does not necessarily constitute an integrated 24hrMB approach. Such frameworks conceptualize sleep, sedentary behavior, and physical activity as interdependent components of a finite 24-hour period, whereby time allocated to one behavior necessarily influences the time available for others [11]. Accordingly, future research would benefit not only from assessing a broader range of movement behaviors concurrently but also from considering analytical approaches capable of examining their interdependence and potential behavioral trade-offs [9-11]. Overall, the findings indicate that the frontline movement behavior literature remains substantially weighted toward sleep, with comparatively limited evidence addressing physical activity, sedentary behavior, or the 3 behaviors collectively. Expanding research beyond isolated movement behaviors may provide a more comprehensive account of daily behavioral patterns and their relationship with mental health in frontline occupational contexts.

### Mental Health Domains and Evidence Concentrations

Mental health research was concentrated around stress, depression, anxiety, and burnout. PTSD or trauma, fatigue, suicidality, and other mental health domains were less frequently represented, while well-being was examined less often than the principal distress-related domains. This concentration is consistent with the substantial psychological demands associated with frontline work, including high occupational demands, irregular working patterns, and potential exposure to distressing or traumatic events [1]. However, it also indicates that current understanding of mental health in relation to movement behaviors is shaped predominantly by a relatively small number of commonly examined psychological domains. Evidence was also unevenly distributed across combinations of movement behaviors and mental health domains. The greatest concentrations involved sleep in relation to stress (n=248), depression (n=226), and anxiety (n=208), with sleep also frequently examined alongside burnout (n=130) and well-being (n=106). Comparatively fewer studies examined mental health domains alongside physical activity, while evidence concerning sedentary behavior was particularly limited across all mental health domains. Therefore, despite the substantial overall volume of literature identified, the depth of evidence varies considerably across movement behavior–mental health combinations, with much of the existing research concentrated around sleep and common indicators of psychological distress.

### Occupational and Contextual Imbalances

Health care workers accounted for the substantial majority of the evidence base, representing 76.3% (468/613) of the included studies. In comparison, considerably fewer studies examined police and law enforcement personnel (n=55, 9.0%), firefighters (n=42, 6.9%), paramedics and emergency medical services personnel (n=25, 4.1%), or mixed frontline worker populations (n=23, 3.8%). This occupational concentration is important because frontline roles differ considerably in their operational demands, work schedules, physical requirements, exposure to potentially traumatic events, and opportunities for rest and recovery [1,6,33,44]. For example, police work may combine prolonged sedentary periods during vehicle-based duties, surveillance, or reporting with intermittent periods of high physical and operational demand, whereas firefighting and emergency medical roles may involve different patterns of acute physical exertion, lifting, equipment carriage, and recovery [45-47]. Such occupational differences may produce distinct movement behavior profiles. Consequently, an evidence base dominated by health care populations may provide a limited understanding of how movement behaviors and mental health are represented across the wider frontline workforce.

The temporal and contextual distribution of the literature may partly explain this occupational concentration. More than four-fifths of included studies were published from 2020 onward, and 175 (28.5%) studies were conducted specifically within a pandemic context. The rapid expansion of research during this period coincided with increased attention to the health and well-being of frontline health care professionals during the COVID-19 pandemic [48,49]. While this substantially expanded the available evidence concerning frontline worker well-being, it may also have contributed to the predominance of health care populations within the literature. Nevertheless, most studies (n=430, 70.1%) were conducted under routine or noncrisis conditions, with comparatively few examining other crisis contexts (n=6, 1.0%) or natural disasters (n=2, 0.3%). Greater occupational diversity within future research would therefore strengthen understanding of movement behaviors and mental health across different frontline settings. Police, firefighters, emergency medical personnel, and other frontline groups may experience distinct combinations of occupational movement, shift work, operational stressors, and recovery opportunities. Expanding research across these populations, and across both routine and crisis contexts, would provide a more comprehensive representation of the frontline workforce and enable greater consideration of occupational and contextual variation [6,45-47].

### Methodological and Measurement Considerations

The evidence base was characterized by a strong predominance of cross-sectional research, with 451 (73.6%) studies using cross-sectional designs, compared with 71 (11.6%) longitudinal and 54 (8.8%) intervention studies. Similarly, most studies were association-focused (n=526, 85.8%), while comparatively few focused on intervention (n=68, 11.1%) or monitoring (n=19, 3.1%). Cross-sectional studies are valuable for characterizing patterns and identifying potential relationships within frontline populations; however, they provide limited insight into temporal relationships or how movement behaviors and mental health may change together over time [43]. The comparatively small longitudinal and intervention literature therefore limits understanding of the temporal dynamics of movement behaviors and mental health and of whether modifying movement behaviors may contribute to changes in mental health domains. Measurement approaches showed a similarly pronounced imbalance. Self-report was the primary movement measurement approach in 533 (86.9%) studies, while 71 (11.6%) combined self-report and objective methods. Only 9 (1.5%) studies relied exclusively on objective movement measurement approaches, including wearable devices, accelerometry, or actigraphy. Although self-report measures provide practical and scalable methods for assessing movement behaviors across large and occupationally diverse samples, comparisons with device-based measures demonstrate that estimates can be affected by reporting, recall, and measurement differences [23-25]. Greater incorporation of objective measurement alongside self-report may therefore provide a more detailed characterization of movement patterns within complex and variable frontline working environments.

Importantly, the use of measurement approaches differed across movement behavior domains. Self-report predominated among studies examining sleep (476/538, 88.5%) and physical activity (133/177, 75.1%), while mixed or objective approaches represented a greater proportion of the comparatively small sedentary behavior literature. These differences demonstrate variation in measurement approaches across movement behavior domains. Objective measurement should not necessarily be regarded as a replacement for self-report, as subjective and objective approaches may capture different aspects of movement and sleep [21,23-25]. Rather, appropriately combining these approaches may provide complementary information, particularly where the research question concerns both perceived experiences and objectively measured behavioral patterns. Where objective monitoring is used, attention should also be given to device validity and reporting standardization. Wearable-derived estimates are not necessarily interchangeable across devices because sensor characteristics, body placement, sampling parameters, wear protocols, and processing algorithms can influence derived movement or sleep metrics [50,51]. External validation approaches, including the framework illustrated by Bačić et al [52] and the broader validation considerations identified by Giurgiu et al [51], demonstrate the value of evaluating wearable-derived estimates against suitable comparator measures under clearly specified conditions and considering how sensor characteristics and processing parameters may influence the resulting metrics. Future studies should therefore report device model, placement, monitoring duration, wear protocol, processing procedures, and relevant validation evidence to support reproducibility and interpretation. These findings also have implications for digital monitoring within frontline occupational settings. Wearable and other objective approaches may enable repeated assessment of movement and recovery patterns across shifts and changing occupational demands; however, their value depends on appropriate validation, transparent reporting, and integration with contextual and self-reported information [21,22,37,50-52]. Future digital monitoring research should therefore prioritize validated devices and metrics, clearly specified monitoring protocols, and worker-centered implementation that considers feasibility, acceptability, privacy, and the interpretation of behavioral or physiological signals within their occupational context.

Future research would therefore benefit from greater methodological diversity, including longitudinal and repeated-measures designs, intervention research, and the considered integration of objective and self-report measurement [21,23-25,43]. Such approaches may be particularly valuable in frontline settings, where movement behaviors, occupational demands, work schedules, and opportunities for recovery can vary considerably within individuals across shifts and overtime [1,2,8]. Together, these methodological developments would enable the field to move beyond predominantly cross-sectional descriptions of associations toward a more temporally informed understanding of movement behaviors and mental health among frontline workers [43].

### Implications for Future Research and Practice

The findings identify several priorities for advancing research on movement behaviors and mental health among frontline workers. First, greater adoption of integrated 24hrMB approaches is needed. Rather than examining sleep, physical activity, and sedentary behavior predominantly in isolation, future studies should consider these behaviors concurrently and, where appropriate, apply analytical approaches that account for their interdependence within a finite 24-hour period. Compositional data analysis and isotemporal substitution modeling may be particularly valuable in this regard, as they enable examination of how different allocations of time across movement behaviors relate to health domains [9-11]. Such approaches may be especially relevant in frontline settings, where shift patterns, long working hours, staffing pressures, and operational demands can constrain opportunities for sleep, movement, and recovery [1,6,7]. Second, future research should address the substantial behavioral and occupational gaps identified in the evidence base. Greater attention to sedentary behavior is particularly warranted, alongside continued investigation of physical activity and the combined contribution of multiple movement behaviors. Research should also extend beyond the predominantly health care–based literature to include greater representation of police, firefighters, emergency medical personnel, and other frontline occupational groups. Given the differing operational demands and working patterns across these occupations, greater occupational diversity would help establish a more comprehensive understanding of movement behaviors and mental health across frontline contexts.

Methodologically, greater use of longitudinal, repeated measures, and intervention designs would strengthen understanding of how movement behaviors and mental health vary over time and across changing occupational conditions. Combining objective movement measurement with self-report may also provide complementary information by capturing both behavioral patterns and individuals’ perceived experiences. These approaches may be particularly useful for examining within-person variation across work shifts, rest days, and periods of differing occupational demand. As longitudinal wearable and repeated-measures datasets develop, future research could also examine appropriately validated predictive modeling and AI approaches for identifying patterns associated with changes in movement behaviors, recovery, or mental health [22,37]. Such applications would require robust validation and careful consideration of privacy, acceptability, and ethical implementation within occupational settings [21,37].

The findings also have implications for occupational practice. The concentration of evidence around sleep reinforces the importance of opportunities for adequate sleep and recovery within frontline work [2,13-15], while the broader evidence gaps suggest that occupational well-being strategies should not necessarily consider movement behaviors in isolation [9-11]. However, the current predominance of cross-sectional and association-focused evidence limits conclusions regarding which changes to movement behaviors would improve mental health domains. Accordingly, the present evidence is better positioned to inform priorities for monitoring and future intervention development than to support specific behavioral prescriptions. Future interventions should also recognize that opportunities to modify sleep, physical activity, and sedentary behavior may be constrained by shift schedules, staffing arrangements, workload, and other organizational factors, highlighting the importance of both individual- and organizational-level approaches [1,2,6-8]. Overall, this review uniquely maps where evidence on movement behaviors and mental health among frontline workers is concentrated and, importantly, highlights the relative absence of research adopting an integrated 24hrMB perspective.

### Strengths and Limitations

This review has several strengths. A comprehensive search strategy was applied across 6 electronic databases and updated on July 30, 2026, to capture recently published evidence, resulting in a large evidence base of 613 studies. Screening procedures incorporated independent assessment of subsets of records by additional reviewers across screening stages, with disagreements resolved through discussion and consensus. Data charting was completed for all included studies by 1 reviewer (AS), with independent charting of random subsets by additional reviewers (GH-B and EC) and 100% agreement within the overlapping sample. The use of non–mutually exclusive coding for movement behaviors and mental health domains enabled studies examining multiple domains to contribute to each relevant category, providing a more detailed representation of the breadth and overlap of the evidence base. Evidence mapping further enabled identification of areas of research concentration and relative scarcity across movement behavior and mental health domains. Stakeholder consultation contributed to refinement of the review questions and eligibility criteria and supported interpretation of the findings and evidence gaps. The review followed JBI guidance for scoping reviews, was reported in accordance with PRISMA-ScR recommendations, and was guided by a previously published protocol, supporting methodological transparency and reproducibility [29,30,32].

Several limitations should also be considered. First, gray literature, citation searching, and other supplementary source searching were not undertaken. The review therefore primarily represents evidence indexed in the 6 searched databases and may not capture relevant unpublished, nonindexed, or otherwise unretrieved studies. Second, formal critical appraisal of individual studies was not undertaken. Critical appraisal is not a mandatory component of scoping review methodology where the objective is to map the extent, characteristics, and distribution of an evidence base rather than to determine intervention effectiveness or estimate the strength or certainty of effects [29]. Accordingly, the findings describe the volume and distribution of research and should not be interpreted as indicating the methodological quality or strength of evidence within individual domains. Third, substantial heterogeneity existed across the included literature in occupational populations, movement behaviors, mental health constructs, study designs, and measurement approaches. Although this breadth was appropriate to the scoping review objectives, it limits direct comparison across studies and means that studies contributing to the same broad domain may have operationalized constructs differently.

The mental health categories were iteratively refined during charting to accommodate the range of constructs encountered, and some domains grouped within the broader “other mental health” category were necessarily heterogeneous. Similarly, broad measurement categories were used to map movement measurement approaches and do not capture all differences in instruments, devices, protocols, or measurement quality. The predominance of cross-sectional and self-report methodologies within the evidence base limits causal inference and understanding of within-person behavioral variability over time [23-25,43]. The distribution of the underlying evidence base should be considered when interpreting the review findings. Research was disproportionately concentrated within health care populations, sleep-focused studies, cross-sectional designs, and self-report measurement approaches. Consequently, the mapped evidence does not represent all frontline occupations, movement behaviors, or methodological approaches equally. Furthermore, because this review mapped the presence and distribution of movement behavior and mental health domains rather than synthesizing the direction, magnitude, or statistical significance of associations, the findings should not be interpreted as evidence that particular movement behaviors cause, protect against, or worsen specific mental health domains. Finally, because wearable technologies, psychophysiological monitoring approaches, and digital health applications continue to evolve rapidly, the findings should be interpreted as reflecting a developing evidence base rather than a static body of literature [21,22,37].

### Conclusions

This scoping review identified a large but unevenly distributed evidence base examining movement behaviors and mental health among frontline workers. Research was concentrated on health care populations, sleep-focused studies, cross-sectional and association-focused designs, and self-report measurement approaches. Physical activity, sedentary behavior, integrated 24hrMB approaches, non–health care frontline occupations, and longitudinal and objective methodologies remained underrepresented. Evidence was also concentrated around stress, depression, and anxiety, particularly in relation to sleep. By mapping movement behavior and mental health domains as overlapping rather than mutually exclusive categories, this review provides a detailed representation of where evidence is concentrated and where substantive behavioral, occupational, and methodological gaps remain. The present evidence is better suited to informing priorities for occupational monitoring and future intervention development than to supporting specific behavioral prescriptions. Future research should broaden the behavioral and occupational scope of the field and examine the interdependence of movement behaviors within constrained occupational time budgets. Developing this evidence base may support a more comprehensive understanding of how movement behaviors, occupational demands, and mental health intersect across the 24-hour day.

## Supplementary material

10.2196/106002 Multimedia Appendix 1 Complete database search strategies.

10.2196/106002 Checklist 1 PRISMA-ScR checklist.

10.2196/106002 Checklist 2 PRISMA-S checklist.

## Abbreviations

JBI: Joanna Briggs Institute
PRISMA-S: Preferred Reporting Items for Systematic Reviews and Meta-Analyses literature search extension
PRISMA-ScR: Preferred Reporting Items for Systematic Reviews and Meta-Analyses Extension for Scoping Reviews
PTSD: posttraumatic stress disorder
24hrMB: 24-hour movement behavior

## References

[1] Cogan N (2026). Persistent traumatic stress exposure: rethinking PTSD for frontline workers. Healthcare (Basel).

[2] Ganesan S, Magee M, Stone JE (2019). The impact of shift work on sleep, alertness and performance in healthcare workers. Sci Rep.

[3] Pappa S, Ntella V, Giannakas T, Giannakoulis VG, Papoutsi E, Katsaounou P (2020). Prevalence of depression, anxiety, and insomnia among healthcare workers during the COVID-19 pandemic: a systematic review and meta-analysis. Brain Behav Immun.

[4] Luan R, Pu W, Dai L, Yang R, Wang P (2020). Comparison of psychological stress levels and associated factors among healthcare workers, frontline workers, and the general public during the novel coronavirus pandemic. Front Psychiatry.

[5] Blasco-Belled A, Tejada-Gallardo C, Fatsini-Prats M, Alsinet C (2022). Mental health among the general population and healthcare workers during the COVID-19 pandemic: a meta-analysis of well-being and psychological distress prevalence. Curr Psychol.

[6] Maglione MA, Chen C, Bialas A (2022). Stress control for military, law enforcement, and first responders: a systematic review. RAND Health Q.

[7] Sumner RC, Kinsella EL (2021). “It’s Like a Kick in the Teeth”: the emergence of novel predictors of burnout in frontline workers during Covid-19. Front Psychol.

[8] Greenberg N, Docherty M, Gnanapragasam S, Wessely S (2020). Managing mental health challenges faced by healthcare workers during Covid-19 pandemic. BMJ.

[9] Ross R, Chaput JP, Giangregorio LM (2020). Canadian 24-hour movement guidelines for adults aged 18-64 years and adults aged 65 years or older: an integration of physical activity, sedentary behaviour, and sleep. Appl Physiol Nutr Metab.

[10] Bull FC, Al-Ansari SS, Biddle S (2020). World Health Organization 2020 guidelines on physical activity and sedentary behaviour. Br J Sports Med.

[11] Brown DMY, Burkart S, Groves CI (2024). A systematic review of research reporting practices in observational studies examining associations between 24-h movement behaviors and indicators of health using compositional data analysis. J Act Sedentary Sleep Behav.

[12] Teychenne M, Ball K, Salmon J (2010). Sedentary behavior and depression among adults: a review. Int J Behav Med.

[13] Furihata R, Saitoh K, Suzuki M (2020). A composite measure of sleep health is associated with symptoms of depression among Japanese female hospital nurses. Compr Psychiatry.

[14] Van Nguyen T, Liu HE (2022). A cross-sectional study on sleep disturbances and associated factors among nurses. BMC Psychiatry.

[15] Taouk L, Farrow VA, Schulkin J (2018). Amount and quality of sleep: exploring the role of stress and work experience in a sample of obstetrician-gynecologists. J Psychosom Obstet Gynaecol.

[16] Meckes SJ, McDonald MA, Lancaster CL (2021). Association between physical activity and mental health among first responders with different service roles. Psychol Trauma.

[17] Bernhard JC, Tornquist L, Marques CT, Peroza LR (2024). Association between physical activity and mental health in health professionals in the context of the pandemic: a study in southern Brazil. J Occup Environ Med.

[18] Wolff MB, O’Connor PJ, Wilson MG, Gay JL (2021). Associations between occupational and leisure-time physical activity with employee stress, burnout and well-being among healthcare industry workers. Am J Health Promot.

[19] Ellingson LD, Meyer JD, Shook RP (2018). Changes in sedentary time are associated with changes in mental wellbeing over 1 year in young adults. Prev Med Rep.

[20] Tsou MT (2022). Influence of prolonged visual display terminal use on physical and mental conditions among health care workers at tertiary hospitals, Taiwan. Int J Environ Res Public Health.

[21] Barac M, Scaletty S, Hassett LC (2024). Wearable technologies for detecting burnout and well-being in health care professionals: scoping review. J Med Internet Res.

[22] Pataca AO, Zdravevski E, Coelho PJ (2025). Use of machine learning for predicting stress episodes based on wearable sensor data: a systematic review. Comput Biol Med.

[23] Prince SA, Adamo KB, Hamel ME, Hardt J, Connor Gorber S, Tremblay M (2008). A comparison of direct versus self-report measures for assessing physical activity in adults: a systematic review. Int J Behav Nutr Phys Act.

[24] Prince SA, Cardilli L, Reed JL (2020). A comparison of self-reported and device measured sedentary behaviour in adults: a systematic review and meta-analysis. Int J Behav Nutr Phys Act.

[25] Girschik J, Fritschi L, Heyworth J, Waters F (2012). Validation of self-reported sleep against actigraphy. J Epidemiol.

[26] Kane L, Powell D, Martin KR, Rees C, Curran J, Ball D (2025). Continuous heart rate variability monitoring, stress and recovery in doctors: a systematic review and meta-analysis. Occup Med (Lond).

[27] Kim HG, Cheon EJ, Bai DS, Lee YH, Koo BH (2018). Stress and heart rate variability: a meta-analysis and review of the literature. Psychiatry Investig.

[28] Samson C, Koh A (2020). Stress monitoring and recent advancements in wearable biosensors. Front Bioeng Biotechnol.

[29] Peters MDJ, Marnie C, Tricco AC (2020). Updated methodological guidance for the conduct of scoping reviews. JBI Evid Synth.

[30] Tricco AC, Lillie E, Zarin W (2018). PRISMA Extension for Scoping Reviews (PRISMA-ScR): checklist and explanation. Ann Intern Med.

[31] Gottlieb M, Haas MRC, Daniel M, Chan TM (2021). The scoping review: a flexible, inclusive, and iterative approach to knowledge synthesis. AEM Educ Train.

[32] Skovgaard A, Kirk A, Cogan N (2026). Examination of relationships between 24-hour movement behaviors and mental health outcomes in frontline workers: protocol for a scoping review. JMIR Res Protoc.

[33] Blau FD, Koebe J, Meyerhofer PA (2021). Who are the essential and frontline workers?. Bus Econ.

[34] Arksey H, O’Malley L (2005). Scoping studies: towards a methodological framework. Int J Soc Res Methodol.

[35] Mak S, Thomas A (2022). Steps for conducting a scoping review. J Grad Med Educ.

[36] Kang HS, Exworthy M (2022). Wearing the future-wearables to empower users to take greater responsibility for their health and care: scoping review. JMIR Mhealth Uhealth.

[37] Piwek L, Ellis DA, Andrews S, Joinson A (2016). The rise of consumer health wearables: promises and barriers. PLoS Med.

[38] Rethlefsen ML, Kirtley S, Waffenschmidt S (2021). PRISMA-S: an extension to the PRISMA statement for reporting literature searches in systematic reviews. Syst Rev.

[39] (2025). Building ideas, one citation at a time. EndNote.

[40] Ouzzani M, Hammady H, Fedorowicz Z, Elmagarmid A (2016). Rayyan-a web and mobile app for systematic reviews. Syst Rev.

[41] Kastner M, Tricco AC, Soobiah C (2012). What is the most appropriate knowledge synthesis method to conduct a review? Protocol for a scoping review. BMC Med Res Methodol.

[42] Hadie SNH (2024). ABC of a scoping review: a simplified JBI scoping review guideline. EIMJ.

[43] Siddaway AP, Wood AM, Hedges LV (2019). How to do a systematic review: a best practice guide for conducting and reporting narrative reviews, meta-analyses, and meta-syntheses. Annu Rev Psychol.

[44] Spicksley DK, Franklin DE (2023). Who works on the “frontline”? Comparing constructions of “frontline” work before and during the COVID-19 pandemic. Appl Corpus Linguistics.

[45] Granholm Valmari E, Ghazinour M, Nygren U, Gilenstam K (2023). A systematic review of lifestyle and health among patrolling police officers. Scand J Occup Ther.

[46] Coffey B, MacPhee R, Socha D, Fischer SL (2016). A physical demands description of paramedic work in Canada. Int J Ind Ergon.

[47] dos Santos ML, Lockie RG, Orr R (2025). The metabolic demand of firefighting: a systematic review. Physiologia.

[48] (2022). World failing in “our duty of care” to protect mental health and well-being of health and care workers, finds report on impact of COVID-19. World Health Organization.

[49] Gould LJ, Angwin E, Powell R, Lawrance E (2024). Healthcare workers’ mental health during the COVID-19 pandemic: a qualitative analysis of a text message-based NHS workforce support line. BMJ Open.

[50] Stangl FJ, Riedl R (2022). Measurement of heart rate and heart rate variability with wearable devices: a systematic review. https://aisel.aisnet.org/wi2022/hci/hci/15/.

[51] Giurgiu M, Timm I, Becker M (2022). Quality evaluation of free-living validation studies for the assessment of 24-hour physical behavior in adults via wearables: systematic review. JMIR Mhealth Uhealth.

[52] Bačić B, Feng C, Li W (2024). JY61 IMU sensor external validity: a framework for advanced pedometer algorithm personalisation. ISBS Proc Arch.

