# Supplementary material for: Mapping Movement Behaviors and Mental Health Among Frontline Workers: Scoping Review
Source: JMIR Public Health Surveill. 2026 Oct 5;12:e106002. doi: 10.2196/106002 (PMC13637985; doi:10.2196/106002)
Supplement: Supplementary file 1 [file publichealth-v12-e106002-s001.docx]

**Multimedia Appendix 1. Complete Database Search Strategies**

Searches were conducted across six electronic databases: APA PsycINFO, CINAHL, MEDLINE, SPORTDiscus, Scopus, and PubMed. The original searches were conducted on 19 November 2025, with updated searches conducted on 30 July 2026. Search strategies combined terms relating to (1) frontline workers and high-stress occupations, (2) movement behaviours and associated monitoring terminology, and (3) mental health and psychological wellbeing, with an exclusion block relating to clinical, patient, and military populations. Database-specific syntax, fields, and filters were applied as appropriate. Where the original database search history was retained, the strategy is reproduced verbatim. For databases for which the exported search history was not retained, the strategy below has been reconstructed from contemporaneous search records documenting the search terms, fields, filters, and retrieval counts used.

## **Original Search**

## **S1. APA PsycINFO**

**Database:** APA PsycINFO
**Platform:** EBSCOhost
**Date searched:** 19 November 2025
**Search fields:** Title and abstract
**Limits/filters:** Peer reviewed; English language; publication years 2000–2025; human population; adult age groups
**Search options:** Find all search terms; Apply equivalent subjects
**Records retrieved:** 1,688

**Search strategy:**

["Frontline worker*" OR "Healthcare worker*" OR "hospital staff" OR "medical staff" OR Nurse* OR Midwife* OR physician* OR doctor* OR surgeon* OR "health professional*" OR physiotherapist* OR radiographer* OR "speech therapist*" OR "psychiatric nurse*" OR "nursing assistant*" OR "healthcare assistant*" OR "residential care worker*" OR "care worker*" OR "hospice worker*" OR "support worker*" OR "Occupational stress worker*" OR "Emergency responder*" OR "First responder*" OR Paramedic* OR "Community paramedic*" OR Firefighter* OR Lifeguard* OR "Search and rescue worker*" OR "Rescue worker*" OR lifesaver* OR Police OR "police officer*" OR "law enforcement" OR "Correctional officer*" OR "prison officer*" OR "Crisis worker*" OR "Crisis hotline worker*" OR "Child protection worker*" OR "Youth worker*" OR "Substance misuse worker*" OR "Disaster relief worker*" OR "Disaster response worker*" OR "Humanitarian worker*" OR "Ambulance staff" OR "emergency medical technician*" OR EMT* OR "Emergency call handler*" OR dispatch* OR "Call handler*" OR "Critical worker*" OR "Essential worker*"]

**AND**

["24-hour movement" OR "24 hour movement" OR "24h movement" OR "movement behaviour*" OR "movement behavior*" OR "movement pattern*" OR "activity pattern*" OR "daily activity*" OR "total physical activity" OR "physical activity" OR "active behaviour*" OR "active behavior*" OR "activity level*" OR "light physical activity" OR LPA OR "moderate-to-vigorous physical activity" OR MVPA OR "sedentary behaviour" OR "sedentary behavior" OR "sedentary lifestyle" OR "sedentary time" OR "sitting time" OR "sitting behaviour*" OR "sitting behavior*" OR inactivity OR "step count*" OR "energy expenditure" OR EE OR sleep* OR SOL OR WASO OR "sleep-wake pattern*" OR "circadian rhythm*" OR rest OR napping OR "rest-activity rhythm*" OR "heart rate" OR HR OR "heart rate variability" OR HRV OR "blood pressure" OR BP OR "cardiac output" OR "electrodermal activity" OR "galvanic skin response" OR GSR OR "skin conductance" OR "skin temperature" OR "vagal tone" OR "autonomic function*" OR "autonomic nervous system measure*" OR "respiratory rate" OR "breathing rate" OR respiration OR "respiratory variability" OR actigraphy OR accelerometry OR "wearable sensor*" OR "fitness tracker*" OR smartwatch* OR "activity monitor*" OR "accelerometer-based monitor*" OR "sensor-based assessment*" OR "motion sensor*" OR "physiological monitor*" OR "biometric monitor*" OR biosensor* OR "physiological signal*" OR "biometric signal*" OR "electrocardiogram" OR ECG OR EKG OR "electromyography" OR EMG OR "electroencephalography" OR EEG OR cortisol OR "salivary cortisol" OR "stress hormone*" OR "pupillometry" OR "pupil dilation" OR "stress response marker*"]

**AND**

["mental health" OR stress OR burnout OR "post-traumatic stress" OR trauma OR "secondary traumatic stress" OR STS OR fatigue OR "moral injury" OR "critical incident stress" OR "psychological distress" OR anxiety OR depression OR "depressive symptom*" OR "anxiety symptom*" OR "emotional dysregulation" OR "affect regulation" OR "self-regulation" OR wellbeing OR "well-being" OR "well being" OR "psychological wellbeing" OR "mental wellbeing" OR "quality of life" OR QoL OR "life satisfaction" OR resilience OR coping OR "coping strategies"]

**NOT**

[patient* OR inpatient* OR outpatient* OR "chronic disease*" OR cancer OR diabetes OR "cardiovascular disease*" OR "neurological disorder*" OR "rehabilitation patient*" OR "hospital patient*" OR "psychiatric patient*" OR "treatment-seeking" OR "pharmacological treatment" OR "psychiatric treatment*" OR "hospitalised" OR "nursing home resident*" OR "care home resident*" OR military OR veteran* OR army OR navy OR airforce OR "armed forces" OR soldier*]

## **Updated search**

**Date searched:** 30 July 2026
**Search fields:** Title and abstract
**Date restriction:** November 2025–July 2026
**Other limits/filters:** Same as the original search
**Search terms:** Same search strategy as the original search
 **Records retrieved:** 49

**Update procedure:** The original search strategy was rerun without modification to the search terms. The publication-date restriction was changed to November 2025-July 2026 to identify records published since the original search.

## **Original Search**

## **S2. CINAHL**

**Database:** CINAHL
 **Platform:** EBSCOhost
 **Date searched:** 19 November 2025
 **Search fields:** Title and abstract
 **Limits/filters:** Peer reviewed; English language; human population; publication date 1 January 2000–31 December 2025; adult age groups
 **Search options:** Find all search terms; Apply equivalent subjects
 **Records retrieved:** 1,197

**Search strategy:**

["Frontline worker*" OR "Healthcare worker*" OR "hospital staff" OR "medical staff" OR Nurse* OR Midwife* OR physician* OR doctor* OR surgeon* OR "health professional*" OR physiotherapist* OR radiographer* OR "speech therapist*" OR "psychiatric nurse*" OR "nursing assistant*" OR "healthcare assistant*" OR "residential care worker*" OR "care worker*" OR "hospice worker*" OR "support worker*" OR "Occupational stress worker*" OR "Emergency responder*" OR "First responder*" OR Paramedic* OR "Community paramedic*" OR Firefighter* OR Lifeguard* OR "Search and rescue worker*" OR "Rescue worker*" OR lifesaver* OR Police OR "police officer*" OR "law enforcement" OR "Correctional officer*" OR "prison officer*" OR "Crisis worker*" OR "Crisis hotline worker*" OR "Child protection worker*" OR "Youth worker*" OR "Substance misuse worker*" OR "Disaster relief worker*" OR "Disaster response worker*" OR "Humanitarian worker*" OR "Ambulance staff" OR "emergency medical technician*" OR EMT* OR "Emergency call handler*" OR dispatch* OR "Call handler*" OR "Critical worker*" OR "Essential worker*"]

**AND**

["24-hour movement" OR "24 hour movement" OR "24h movement" OR "movement behaviour*" OR "movement behavior*" OR "movement pattern*" OR "activity pattern*" OR "daily activity*" OR "total physical activity" OR "physical activity" OR "active behaviour*" OR "active behavior*" OR "activity level*" OR "light physical activity" OR LPA OR "moderate-to-vigorous physical activity" OR MVPA OR "sedentary behaviour" OR "sedentary behavior" OR "sedentary lifestyle" OR "sedentary time" OR "sitting time" OR "sitting behaviour*" OR "sitting behavior*" OR inactivity OR "step count*" OR "energy expenditure" OR EE OR sleep* OR SOL OR WASO OR "sleep-wake pattern*" OR "circadian rhythm*" OR rest OR napping OR "rest-activity rhythm*" OR "heart rate" OR HR OR "heart rate variability" OR HRV OR "blood pressure" OR BP OR "cardiac output" OR "electrodermal activity" OR "galvanic skin response" OR GSR OR "skin conductance" OR "skin temperature" OR "vagal tone" OR "autonomic function*" OR "autonomic nervous system measure*" OR "respiratory rate" OR "breathing rate" OR respiration OR "respiratory variability" OR actigraphy OR accelerometry OR "wearable sensor*" OR "fitness tracker*" OR smartwatch* OR "activity monitor*" OR "accelerometer-based monitor*" OR "sensor-based assessment*" OR "motion sensor*" OR "physiological monitor*" OR "biometric monitor*" OR biosensor* OR "physiological signal*" OR "biometric signal*" OR "electrocardiogram" OR ECG OR EKG OR "electromyography" OR EMG OR "electroencephalography" OR EEG OR cortisol OR "salivary cortisol" OR "stress hormone*" OR "pupillometry" OR "pupil dilation" OR "stress response marker*"]

**AND**

["mental health" OR stress OR burnout OR "post-traumatic stress" OR trauma OR "secondary traumatic stress" OR STS OR fatigue OR "moral injury" OR "critical incident stress" OR "psychological distress" OR anxiety OR depression OR "depressive symptom*" OR "anxiety symptom*" OR "emotional dysregulation" OR "affect regulation" OR "self-regulation" OR wellbeing OR "well-being" OR "well being" OR "psychological wellbeing" OR "mental wellbeing" OR "quality of life" OR QoL OR "life satisfaction" OR resilience OR coping OR "coping strategies"]

**NOT**

[patient* OR inpatient* OR outpatient* OR "chronic disease*" OR cancer OR diabetes OR "cardiovascular disease*" OR "neurological disorder*" OR "rehabilitation patient*" OR "hospital patient*" OR "psychiatric patient*" OR "treatment-seeking" OR "pharmacological treatment" OR "psychiatric treatment*" OR "hospitalised" OR "nursing home resident*" OR "care home resident*" OR military OR veteran* OR army OR navy OR airforce OR "armed forces" OR soldier*]

## **Updated search**

**Date searched:** 30 July 2026
 **Search fields:** Title and abstract
 **Date restriction:** November 2025–July 2026
 **Other limits/filters:** Same as the original search
 **Search terms:** Same search strategy as the original search
 **Records retrieved:** 79

**Update procedure:** The original search strategy was rerun without modification to the search terms. The publication-date restriction was changed to November 2025-July 2026 to identify records published since the original search.

## **Original Search**

## **S3. MEDLINE**

**Database:** MEDLINE
 **Platform:** EBSCOhost
 **Date searched:** 19 November 2025
 **Search fields:** Title and abstract
 **Limits/filters:** Peer reviewed; English language; human population; publication date 1 January 2000–31 December 2025; adult age groups (19+ years)
 **Search options:** Find all search terms; Apply equivalent subjects
 **Records retrieved:** 2,653

**Search strategy:**

["Frontline worker*" OR "Healthcare worker*" OR "hospital staff" OR "medical staff" OR Nurse* OR Midwife* OR physician* OR doctor* OR surgeon* OR "health professional*" OR physiotherapist* OR radiographer* OR "speech therapist*" OR "psychiatric nurse*" OR "nursing assistant*" OR "healthcare assistant*" OR "residential care worker*" OR "care worker*" OR "hospice worker*" OR "support worker*" OR "Occupational stress worker*" OR "Emergency responder*" OR "First responder*" OR Paramedic* OR "Community paramedic*" OR Firefighter* OR Lifeguard* OR "Search and rescue worker*" OR "Rescue worker*" OR lifesaver* OR Police OR "police officer*" OR "law enforcement" OR "Correctional officer*" OR "prison officer*" OR "Crisis worker*" OR "Crisis hotline worker*" OR "Child protection worker*" OR "Youth worker*" OR "Substance misuse worker*" OR "Disaster relief worker*" OR "Disaster response worker*" OR "Humanitarian worker*" OR "Ambulance staff" OR "emergency medical technician*" OR EMT* OR "Emergency call handler*" OR dispatch* OR "Call handler*" OR "Critical worker*" OR "Essential worker*"]

**AND**

["24-hour movement" OR "24 hour movement" OR "24h movement" OR "movement behaviour*" OR "movement behavior*" OR "movement pattern*" OR "activity pattern*" OR "daily activity*" OR "total physical activity" OR "physical activity" OR "active behaviour*" OR "active behavior*" OR "activity level*" OR "light physical activity" OR LPA OR "moderate-to-vigorous physical activity" OR MVPA OR "sedentary behaviour" OR "sedentary behavior" OR "sedentary lifestyle" OR "sedentary time" OR "sitting time" OR "sitting behaviour*" OR "sitting behavior*" OR inactivity OR "step count*" OR "energy expenditure" OR EE OR sleep* OR SOL OR WASO OR "sleep-wake pattern*" OR "circadian rhythm*" OR rest OR napping OR "rest-activity rhythm*" OR "heart rate" OR HR OR "heart rate variability" OR HRV OR "blood pressure" OR BP OR "cardiac output" OR "electrodermal activity" OR "galvanic skin response" OR GSR OR "skin conductance" OR "skin temperature" OR "vagal tone" OR "autonomic function*" OR "autonomic nervous system measure*" OR "respiratory rate" OR "breathing rate" OR respiration OR "respiratory variability" OR actigraphy OR accelerometry OR "wearable sensor*" OR "fitness tracker*" OR smartwatch* OR "activity monitor*" OR "accelerometer-based monitor*" OR "sensor-based assessment*" OR "motion sensor*" OR "physiological monitor*" OR "biometric monitor*" OR biosensor* OR "physiological signal*" OR "biometric signal*" OR "electrocardiogram" OR ECG OR EKG OR "electromyography" OR EMG OR "electroencephalography" OR EEG OR cortisol OR "salivary cortisol" OR "stress hormone*" OR "pupillometry" OR "pupil dilation" OR "stress response marker*"]

**AND**

["mental health" OR stress OR burnout OR "post-traumatic stress" OR trauma OR "secondary traumatic stress" OR STS OR fatigue OR "moral injury" OR "critical incident stress" OR "psychological distress" OR anxiety OR depression OR "depressive symptom*" OR "anxiety symptom*" OR "emotional dysregulation" OR "affect regulation" OR "self-regulation" OR wellbeing OR "well-being" OR "well being" OR "psychological wellbeing" OR "mental wellbeing" OR "quality of life" OR QoL OR "life satisfaction" OR resilience OR coping OR "coping strategies"]

**NOT**

[patient* OR inpatient* OR outpatient* OR "chronic disease*" OR cancer OR diabetes OR "cardiovascular disease*" OR "neurological disorder*" OR "rehabilitation patient*" OR "hospital patient*" OR "psychiatric patient*" OR "treatment-seeking" OR "pharmacological treatment" OR "psychiatric treatment*" OR "hospitalised" OR "nursing home resident*" OR "care home resident*" OR military OR veteran* OR army OR navy OR airforce OR "armed forces" OR soldier*]

## **Updated search**

**Date searched:** 30 July 2026
 **Search fields:** Title and abstract
 **Date restriction:** November 2025–July 2026
 **Other limits/filters:** Same as the original search
 **Search terms:** Same search strategy as the original search
 **Records retrieved:** 292

**Update procedure:** The original search strategy was rerun without modification to the search terms. The publication-date restriction was changed to November 2025-July 2026 to identify records published since the original search.

## **Original Search**

## **S4. SPORTDiscus**

**Database:** SPORTDiscus
 **Platform:** EBSCOhost
 **Date searched:** 19 November 2025
 **Search fields:** Title and abstract
 **Limits/filters:** Peer reviewed; English language; publication date 1 January 2000–31 December 2025
 **Search options:** Find all search terms; Apply equivalent subjects
 **Records retrieved:** 438

**Search strategy:**

["Frontline worker*" OR "Healthcare worker*" OR "hospital staff" OR "medical staff" OR Nurse* OR Midwife* OR physician* OR doctor* OR surgeon* OR "health professional*" OR physiotherapist* OR radiographer* OR "speech therapist*" OR "psychiatric nurse*" OR "nursing assistant*" OR "healthcare assistant*" OR "residential care worker*" OR "care worker*" OR "hospice worker*" OR "support worker*" OR "Occupational stress worker*" OR "Emergency responder*" OR "First responder*" OR Paramedic* OR "Community paramedic*" OR Firefighter* OR Lifeguard* OR "Search and rescue worker*" OR "Rescue worker*" OR lifesaver* OR Police OR "police officer*" OR "law enforcement" OR "Correctional officer*" OR "prison officer*" OR "Crisis worker*" OR "Crisis hotline worker*" OR "Child protection worker*" OR "Youth worker*" OR "Substance misuse worker*" OR "Disaster relief worker*" OR "Disaster response worker*" OR "Humanitarian worker*" OR "Ambulance staff" OR "emergency medical technician*" OR EMT* OR "Emergency call handler*" OR dispatch* OR "Call handler*" OR "Critical worker*" OR "Essential worker*"]

**AND**

["24-hour movement" OR "24 hour movement" OR "24h movement" OR "movement behaviour*" OR "movement behavior*" OR "movement pattern*" OR "activity pattern*" OR "daily activity*" OR "total physical activity" OR "physical activity" OR "active behaviour*" OR "active behavior*" OR "activity level*" OR "light physical activity" OR LPA OR "moderate-to-vigorous physical activity" OR MVPA OR "sedentary behaviour" OR "sedentary behavior" OR "sedentary lifestyle" OR "sedentary time" OR "sitting time" OR "sitting behaviour*" OR "sitting behavior*" OR inactivity OR "step count*" OR "energy expenditure" OR EE OR sleep* OR SOL OR WASO OR "sleep-wake pattern*" OR "circadian rhythm*" OR rest OR napping OR "rest-activity rhythm*" OR "heart rate" OR HR OR "heart rate variability" OR HRV OR "blood pressure" OR BP OR "cardiac output" OR "electrodermal activity" OR "galvanic skin response" OR GSR OR "skin conductance" OR "skin temperature" OR "vagal tone" OR "autonomic function*" OR "autonomic nervous system measure*" OR "respiratory rate" OR "breathing rate" OR respiration OR "respiratory variability" OR actigraphy OR accelerometry OR "wearable sensor*" OR "fitness tracker*" OR smartwatch* OR "activity monitor*" OR "accelerometer-based monitor*" OR "sensor-based assessment*" OR "motion sensor*" OR "physiological monitor*" OR "biometric monitor*" OR biosensor* OR "physiological signal*" OR "biometric signal*" OR "electrocardiogram" OR ECG OR EKG OR "electromyography" OR EMG OR "electroencephalography" OR EEG OR cortisol OR "salivary cortisol" OR "stress hormone*" OR "pupillometry" OR "pupil dilation" OR "stress response marker*"]

**AND**

["mental health" OR stress OR burnout OR "post-traumatic stress" OR trauma OR "secondary traumatic stress" OR STS OR fatigue OR "moral injury" OR "critical incident stress" OR "psychological distress" OR anxiety OR depression OR "depressive symptom*" OR "anxiety symptom*" OR "emotional dysregulation" OR "affect regulation" OR "self-regulation" OR wellbeing OR "well-being" OR "well being" OR "psychological wellbeing" OR "mental wellbeing" OR "quality of life" OR QoL OR "life satisfaction" OR resilience OR coping OR "coping strategies"]

**NOT**

[patient* OR inpatient* OR outpatient* OR "chronic disease*" OR cancer OR diabetes OR "cardiovascular disease*" OR "neurological disorder*" OR "rehabilitation patient*" OR "hospital patient*" OR "psychiatric patient*" OR "treatment-seeking" OR "pharmacological treatment" OR "psychiatric treatment*" OR "hospitalised" OR "nursing home resident*" OR "care home resident*" OR military OR veteran* OR army OR navy OR airforce OR "armed forces" OR soldier*]

## **Updated search**

**Date searched:** 30 July 2026
 **Search fields:** Title and abstract
 **Date restriction:** November 2025–July 2026
 **Other limits/filters:** Same as the original search
 **Search terms:** Same search strategy as the original search
 **Records retrieved:** 26

**Update procedure:** The original search strategy was rerun without modification to the search terms. The publication-date restriction was changed to November 2025-July 2026 to identify records published since the original search.

## **Original Search**

## **S5. PubMed**

**Database:** PubMed
 **Platform:** National Library of Medicine
 **Original search date:** 19 November 2025
 **Search fields:** Title and abstract
 **Limits/filters:** English language; human population; adult population; publication date 1 January 2000–31 December 2025
 **Records retrieved:** 2,725

**Search strategy:**

["Frontline worker*" OR "Healthcare worker*" OR "hospital staff" OR "medical staff" OR Nurse* OR Midwife* OR physician* OR doctor* OR surgeon* OR "health professional*" OR physiotherapist* OR radiographer* OR "speech therapist*" OR "psychiatric nurse*" OR "nursing assistant*" OR "healthcare assistant*" OR "residential care worker*" OR "care worker*" OR "hospice worker*" OR "support worker*" OR "Occupational stress worker*" OR "Emergency responder*" OR "First responder*" OR Paramedic* OR "Community paramedic*" OR Firefighter* OR Lifeguard* OR "Search and rescue worker*" OR "Rescue worker*" OR lifesaver* OR Police OR "police officer*" OR "law enforcement" OR "Correctional officer*" OR "prison officer*" OR "Crisis worker*" OR "Crisis hotline worker*" OR "Child protection worker*" OR "Youth worker*" OR "Substance misuse worker*" OR "Disaster relief worker*" OR "Disaster response worker*" OR "Humanitarian worker*" OR "Ambulance staff" OR "emergency medical technician*" OR EMT* OR "Emergency call handler*" OR dispatch* OR "Call handler*" OR "Critical worker*" OR "Essential worker*"]

**AND**

["24-hour movement" OR "24 hour movement" OR "24h movement" OR "movement behaviour*" OR "movement behavior*" OR "movement pattern*" OR "activity pattern*" OR "daily activity*" OR "total physical activity" OR "physical activity" OR "active behaviour*" OR "active behavior*" OR "activity level*" OR "light physical activity" OR LPA OR "moderate-to-vigorous physical activity" OR MVPA OR "sedentary behaviour" OR "sedentary behavior" OR "sedentary lifestyle" OR "sedentary time" OR "sitting time" OR "sitting behaviour*" OR "sitting behavior*" OR inactivity OR "step count*" OR "energy expenditure" OR EE OR sleep* OR SOL OR WASO OR "sleep-wake pattern*" OR "circadian rhythm*" OR rest OR napping OR "rest-activity rhythm*" OR "heart rate" OR HR OR "heart rate variability" OR HRV OR "blood pressure" OR BP OR "cardiac output" OR "electrodermal activity" OR "galvanic skin response" OR GSR OR "skin conductance" OR "skin temperature" OR "vagal tone" OR "autonomic function*" OR "autonomic nervous system measure*" OR "respiratory rate" OR "breathing rate" OR respiration OR "respiratory variability" OR actigraphy OR accelerometry OR "wearable sensor*" OR "fitness tracker*" OR smartwatch* OR "activity monitor*" OR "accelerometer-based monitor*" OR "sensor-based assessment*" OR "motion sensor*" OR "physiological monitor*" OR "biometric monitor*" OR biosensor* OR "physiological signal*" OR "biometric signal*" OR "electrocardiogram" OR ECG OR EKG OR "electromyography" OR EMG OR "electroencephalography" OR EEG OR cortisol OR "salivary cortisol" OR "stress hormone*" OR "pupillometry" OR "pupil dilation" OR "stress response marker*"]

**AND**

["mental health" OR stress OR burnout OR "post-traumatic stress" OR trauma OR "secondary traumatic stress" OR STS OR fatigue OR "moral injury" OR "critical incident stress" OR "psychological distress" OR anxiety OR depression OR "depressive symptom*" OR "anxiety symptom*" OR "emotional dysregulation" OR "affect regulation" OR "self-regulation" OR wellbeing OR "well-being" OR "well being" OR "psychological wellbeing" OR "mental wellbeing" OR "quality of life" OR QoL OR "life satisfaction" OR resilience OR coping OR "coping strategies"]

**NOT**

[patient* OR inpatient* OR outpatient* OR "chronic disease*" OR cancer OR diabetes OR "cardiovascular disease*" OR "neurological disorder*" OR "rehabilitation patient*" OR "hospital patient*" OR "psychiatric patient*" OR "treatment-seeking" OR "pharmacological treatment" OR "psychiatric treatment*" OR "hospitalised" OR "nursing home resident*" OR "care home resident*" OR military OR veteran* OR army OR navy OR airforce OR "armed forces" OR soldier*]

## **Updated search**

**Date searched:** 30 July 2026
 **Search fields:** Title and abstract
 **Date restriction:** November 2025–July 2026
 **Other limits/filters:** Same as the original search
 **Search terms:** Same search strategy as the original search
 **Records retrieved:** 317

**Update procedure:** The original search strategy was rerun without modification to the search terms. The publication-date restriction was changed to November 2025-July 2026 to identify records published since the original search.

## **Original Search**

## **S6. Scopus**

**Database:** Scopus

**Platform:** Elsevier Scopus

**Original search date:** 19 November 2025

**Search fields:** Title, abstract, and keywords

**Limits/filters:** English language; publication years 2000–2025; records indexed with child/adolescent, animal/nonhuman, and pregnancy-related keywords excluded.

**Records retrieved:** 9,368

**Search strategy:**

TITLE-ABS-KEY ( "Frontline worker*" OR "Healthcare worker*" OR "hospital staff" OR "medical staff" OR Nurse* OR Midwife* OR physician* OR doctor* OR surgeon* OR "health professional*" OR physiotherapist* OR radiographer* OR "speech therapist*" OR "psychiatric nurse*" OR "nursing assistant*" OR "healthcare assistant*" OR "residential care worker*" OR "care worker*" OR "hospice worker*" OR "support worker*" OR "Occupational stress worker*" OR "Emergency responder*" OR "First responder*" OR Paramedic* OR "Community paramedic*" OR Firefighter* OR Lifeguard* OR "Search and rescue worker*" OR "Rescue worker*" OR lifesaver* OR Police OR "police officer*" OR "law enforcement" OR "Correctional officer*" OR "prison officer*" OR "Crisis worker*" OR "Crisis hotline worker*" OR "Child protection worker*" OR "Youth worker*" OR "Substance misuse worker*" OR "Disaster relief worker*" OR "Disaster response worker*" OR "Humanitarian worker*" OR "Ambulance staff" OR "emergency medical technician*" OR EMT* OR "Emergency call handler*" OR dispatch* OR "Call handler*" OR "Critical worker*" OR "Essential worker*" ) AND TITLE-ABS-KEY ( "24-hour movement" OR "24 hour movement" OR "24h movement" OR "movement behaviour*" OR "movement behavior*" OR "movement pattern*" OR "activity pattern*" OR "daily activity*" OR "total physical activity" OR "physical activity" OR "active behaviour*" OR "active behavior*" OR "activity level*" OR "light physical activity" OR LPA OR "moderate-to-vigorous physical activity" OR MVPA OR "sedentary behaviour" OR "sedentary behavior" OR "sedentary lifestyle" OR "sedentary time" OR "sitting time" OR "sitting behaviour*" OR "sitting behavior*" OR inactivity OR "step count*" OR "energy expenditure" OR EE OR sleep* OR SOL OR WASO OR "sleep-wake pattern*" OR "circadian rhythm*" OR rest OR napping OR "rest-activity rhythm*" OR "heart rate" OR HR OR "heart rate variability" OR HRV OR "blood pressure" OR BP OR "cardiac output" OR "electrodermal activity" OR "galvanic skin response" OR GSR OR "skin conductance" OR "skin temperature" OR "vagal tone" OR "autonomic function*" OR "autonomic nervous system measure*" OR "respiratory rate" OR "breathing rate" OR respiration OR "respiratory variability" OR actigraphy OR accelerometry OR "wearable sensor*" OR "fitness tracker*" OR smartwatch* OR "activity monitor*" OR "accelerometer-based monitor*" OR "sensor-based assessment*" OR "motion sensor*" OR "physiological monitor*" OR "biometric monitor*" OR biosensor* OR "physiological signal*" OR "biometric signal*" OR "electrocardiogram" OR ECG OR EKG OR "electromyography" OR EMG OR "electroencephalography" OR EEG OR cortisol OR "salivary cortisol" OR "stress hormone*" OR "pupillometry" OR "pupil dilation" OR "stress response marker*" ) AND TITLE-ABS-KEY ( "mental health" OR stress OR burnout OR "post-traumatic stress" OR trauma OR "secondary traumatic stress" OR STS OR fatigue OR "moral injury" OR "critical incident stress" OR "psychological distress" OR anxiety OR depression OR "depressive symptom*" OR "anxiety symptom*" OR "emotional dysregulation" OR "affect regulation" OR "self-regulation" OR wellbeing OR "well-being" OR "well being" OR "psychological wellbeing" OR "mental wellbeing" OR "quality of life" OR QoL OR "life satisfaction" OR resilience OR coping OR "coping strategies" ) AND NOT TITLE-ABS-KEY ( patient* OR inpatient* OR outpatient* OR "chronic disease*" OR cancer OR diabetes OR "cardiovascular disease*" OR "neurological disorder*" OR "rehabilitation patient*" OR "hospital patient*" OR "psychiatric patient*" OR "treatment-seeking" OR "pharmacological treatment" OR "psychiatric treatment*" OR "hospitalised" OR "nursing home resident*" OR "care home resident*" OR military OR veteran* OR army OR navy OR airforce OR "armed forces" OR soldier* ) AND ( LIMIT-TO ( PUBYEAR , 2000 ) OR LIMIT-TO ( PUBYEAR , 2001 ) OR LIMIT-TO ( PUBYEAR , 2002 ) OR LIMIT-TO ( PUBYEAR , 2003 ) OR LIMIT-TO ( PUBYEAR , 2004 ) OR LIMIT-TO ( PUBYEAR , 2005 ) OR LIMIT-TO ( PUBYEAR , 2006 ) OR LIMIT-TO ( PUBYEAR , 2007 ) OR LIMIT-TO ( PUBYEAR , 2008 ) OR LIMIT-TO ( PUBYEAR , 2009 ) OR LIMIT-TO ( PUBYEAR , 2010 ) OR LIMIT-TO ( PUBYEAR , 2011 ) OR LIMIT-TO ( PUBYEAR , 2012 ) OR LIMIT-TO ( PUBYEAR , 2013 ) OR LIMIT-TO ( PUBYEAR , 2014 ) OR LIMIT-TO ( PUBYEAR , 2015 ) OR LIMIT-TO ( PUBYEAR , 2016 ) OR LIMIT-TO ( PUBYEAR , 2017 ) OR LIMIT-TO ( PUBYEAR , 2018 ) OR LIMIT-TO ( PUBYEAR , 2019 ) OR LIMIT-TO ( PUBYEAR , 2020 ) OR LIMIT-TO ( PUBYEAR , 2021 ) OR LIMIT-TO ( PUBYEAR , 2022 ) OR LIMIT-TO ( PUBYEAR , 2023 ) OR LIMIT-TO ( PUBYEAR , 2024 ) OR LIMIT-TO ( PUBYEAR , 2025 ) ) AND ( EXCLUDE ( EXACTKEYWORD , "Adolescent" ) OR EXCLUDE ( EXACTKEYWORD , "Child" ) OR EXCLUDE ( EXACTKEYWORD , "Infant" ) OR EXCLUDE ( EXACTKEYWORD , "Preschool Child" ) OR EXCLUDE ( EXACTKEYWORD , "Newborn" ) OR EXCLUDE ( EXACTKEYWORD , "Child, Preschool" ) OR EXCLUDE ( EXACTKEYWORD , "Animals" ) OR EXCLUDE ( EXACTKEYWORD , "Nonhuman" ) OR EXCLUDE ( EXACTKEYWORD , "Pregnancy" ) ) AND ( LIMIT-TO ( LANGUAGE , "English" ) )

## **Updated search**

**Date searched:** 30 July 2026
 **Search fields:** Title, abstract, and keywords
 **Publication-date restriction:** November 2025–July 2026
 **Other limits/filters:** Same as the original search
 **Search terms:** Same search strategy as the original search
 **Records retrieved:** 1,609

**Update procedure:** The original search strategy was rerun without modification to the search terms, with the publication-date restriction updated to identify records published since the original search.
